# In Vivo Antiplaque Effect of Three Edible Toothpastes

**DOI:** 10.4317/medoral.18973

**Published:** 2013-08-29

**Authors:** Susana Rubido, Javier Fernández-Feijoo, Jacobo Limeres, Lucía García-Caballero, María T. Abeleira, Pedro Diz

**Affiliations:** 1 CMB, Grupo de Investigación en Odontología Médico-Quirúrgica (OMEQUI), School of Medicine and Dentistry, Santiago de Compostela University, Spain; 2 MD, DDS, PhD, Grupo de Investigación en Odontología Médico-Quirúrgica (OMEQUI), School of Medicine and Dentistry, Santiago de Compostela University, Spain; 3 DDS, PhD, Grupo de Investigación en Odontología Médico-Quirúrgica (OMEQUI), School of Medicine and Dentistry, Santiago de Compostela University, Spain; 4 MD, DDS, PhD, Associate Prof, Grupo de Investigación en Odontología Médico-Quirúrgica (OMEQUI), School of Medicine and Dentistry, Santiago de Compostela University, Spain

## Abstract

Objectives: The objective of this study was to analyse the antibacterial and antiplaque activity of three edible toothpastes with the widest worldwide distribution: KidScents™, which contains essential oils; Browning B&B™, with medicinal plants; and Wysong Probiodent™, which contains probiotics. Study Design: The study group was formed of twenty healthy volunteers (dental students) with a good oral health status. Using a balanced randomisation system, all volunteers performed toothbrushing with four products (the three edible toothpastes and water) at intervals of one week. Bacterial vitality in the saliva was analysed by epifluorescence microscopy and plaque regrowth was evaluated using the Turesky-Quigley-Hein plaque index. Results: Bacterial vitality in the saliva was significantly higher after toothbrushing with water (positive control) than with the three toothpastes (P=0.002, P=0.003 and P<0.001, respectively). The plaque index was significantly higher after using these three toothpastes than after toothbrushing with water (P=0.047, P=0.032 and P<0.001, respectively). Conclusions: The three edible toothpastes analysed have some antimicrobial activity but favour plaque regrowth.

** Key words:**Edible toothpaste, dental plaque, oral bacteria.

## Introduction

The widespread use of toothpastes, particularly those containing fluoride, was based mainly on their efficacy in the prevention of caries (1). The most common undesirable effect of fluoride is fluorosis and, to minimise this effect, it is recommended that children use very small quantities of toothpaste and spit out the residue (2). However, it is sometimes impossible to prevent a child from swallowing the toothpaste and this has led to marked differences in the proposals of various international dental organizations with regard to the most appropriate choice of toothpaste for children, the age at which to start its prescription and up to what age supervised tooth brushing is recommended (3).

At the age when small children are starting to perform toothbrushing techniques, some have difficulty spitting and they have a tendency to swallow the toothpaste. This has favoured the development of the so-called “edible” toothpastes, which do not contain detergents -and thus do not form foam- or fluoride or other potentially harmful components, and they can therefore be swallowed safely. The elaboration of edible toothpastes became a challenge for the scientists at the North American Space Agency because, in a weightless environment, astronauts found it very difficult to brush their teeth with conventional toothpaste. In the early eighties, Ira L. Shannon, a dentist at the Johnson Space Center associated with the Oral Disease Research Laboratory of the Veterans Administration Hospital in Houston, developed a new type of toothpaste (Nasadent™) for use in a zero gravity environment; that toothpaste did not produce foam and could be swallowed (Patent number: NASA-TM-107998, NAS 1.15:107998). Even though its clinical efficacy and its acceptance by users were comparable with conventional toothpastes, Nasadent™ stopped being marketed some years later. Its principal characteristic was that it eliminated the formation of air bubbles and it was therefore suggested that it could be used in bed-bound patients with difficulty expectorating, tetraplegic patients, patients with neuromuscular degenerative diseases, patients with orofacial paralysis, psychiatric patients and other groups in which assisted toothbrushing may be required.

The composition of modern toothpastes is more complex because they frequently incorporate other constituents that are also beneficial for oral health, reducing calculus formation, extrinsic dental staining, tooth hypersensitivity and halitosis (4). Although in principle their safety and efficacy are guaranteed, some toothpastes are considered cosmetics while others are medicinal prod-ucts; this can lead to difference in marketing regulations (4). Despite this, conventional toothpastes are typically supported by quality clinical trials and rigorous expert reviews (4) though, paradoxically, we have found no publications in the literature that evaluate the efficacy of edible toothpastes. The objective of this study was to analyze the antibacterial and antiplaque activity of three of the edible toothpastes with the widest worldwide distribution.

## Material and Methods

The toothpastes tested in this study were KidScents™ (Young Living Essential Oils, Lehi, UT, USA), Browning B&B™ (Boryung Medience Co Ltd, Seoul, South Korea) and Wysong Probiodent™ (Wysong Corporation, Midland, MI, USA). These toothpastes were selected on the basis of their wide distribution and affordability. The composition of KidScents™ includes calcium carbonate, deionised water, colloidal silver, strawberry aroma, peppermint essential oil (*Mentha piperita*), vegetable glycerin, zinc oxide, xanthan gum, ionic minerals, xylitol, spearmint essential oil (*Mentha spicata*), clove essential oil (*Syzygium aromaticum*), lemon oil (Citrus limon), orange essential oil (*Citrus aurantium*) and Thieves™ essential oil. Browning B&B™ toothpaste contains poloxamer 407, dimethicone, xylitol and natural plant extracts. Wysong Probiodent™ toothpaste includes desiccated sea plankton, trona mineral salts, calcium lactate, birch bark extract, aloe vera, peppermint, potassium citrate, probiotic cultures (*Streptococcus salivarius, Lactobacillus salivarius, Bifidobacterium bifidum, Enterococcus faecium, Lactobacillus acidophilus* and *Lactococcus plantarum*), apple polyphenols, enzymes (amylase, protease and cellulase) and isolated milk proteins.

The study group was formed of twenty healthy volunteers (dental students) with a good oral health status, a minimum of 24 examinable permanent teeth, no evidence of gingivitis or periodontitis (Community Periodontal Index score= 0), and no active caries. The exclusion criteria applied were the following: smoking, dental prosthesis or orthodontic devices, administration of antibiotics or routine use of oral antiseptics in the previous three months and the presence of any systemic disease that could alter the production or composition of the saliva or dental plaque. Tartrectomy was performed on all volunteers before starting the study.

The experiments were started at 9 a.m. and participants were requested not to use any type of toothpaste or mouthwash for 12 hours before the study in order to avoid any possible residual effect of the oral hygiene product that they usually used. By random selection, the volunteers performed toothbrushing with 10 ml of sterile water (positive control), 0.4 ml of KidScents™ toothpaste, 0.4 ml Browning B&B™ toothpaste, or 0.4 ml of Wysong Probiodent™ toothpaste obtained by dissolving 0.7 g of powder in 1 ml of water. After applying sterile water or the selected toothpaste, participants performed toothbrushing for two minutes using a conventional technique, attempting to clean all the tooth surfaces, tongue and mucosas, without wetting the toothbrush in water (except when performing the positive control with sterile water). Without rinsing the toothbrush in water, participants were then given an erythrosine tablet (Plac control®, Dentaid, Barcelona, Spain), which they chewed following the manufacturer’s instructions in order to highlight residual plaque. Using the residual toothpaste on the toothbrush or, in the case of brushing with water, after wetting the toothbrush in water, participants then continued the toothbrushing activity until complete disappearance of the stained dental plaque.

Five minutes after completing toothbrushing, samples of 1 ml of unstimulated saliva were collected using the spitting method (5). To evaluate the antibacterial activity of the toothpastes tested, we analysed the vitality of salivary bacteria using epifluorescence microscopy with the LIVE/DEAD® BacLight™ fluorescence solution (Molecular Probes, Leiden, The Netherlands). This technique has been described in detail and its efficacy has been demonstrated previously (6).

After completing toothbrushing, the participants did not perform any oral hygiene procedure, did not chew gum, did not eat apples or other foods that favour plaque removal and did not drink alcohol for a period of 24 hours in order not to alter dental plaque regrowth. The antiplaque activity of the products was then evaluated by determining the level of newly formed plaque, quantified by visual inspection of erythrosine-stained plaque at six sites per tooth, using the Turesky modification of the Quigley-Hein plaque index (7,8), with a scale from 0 (no plaque) to 5 (plaque covering more than two thirds of the tooth surface).

Using a balanced randomisation system, all volunteers performed toothbrushing with the four products (the three edible tooth-pastes and water) at intervals of one week, during which the participants only used their usual toothpaste and toothbrush.

The statistical analysis was performed with the R software environment (R Development Core Team, 2011, Vienna, Austria). Repeated measure ANOVA was used for inter-toothbrushing comparisons between water and each edible toothpaste and between different toothpastes for each outcome variable (bacterial vitality and the plaque index). Statistical significance was taken as a P value less than 0.05.

This project was approved by the Ethics Committee of the School of Medicine and Dentistry of Santiago de Compostela University. The study was conducted in accordance with the Helsinki Declaration of 1975, as revised in 2000. Informed consent was obtained in writing from all participants in the study.

## Results

The highest levels of bacterial vitality in the saliva were detected after toothbrushing with sterile water (mean=69.28%; range=45%-83.30%). The mean levels of bacterial vitality after toothbrushing with the KidScents™, Browning B&B™ and Wysong Probiodent™ toothpastes were 59.14%, 59.19% and 57.05%, respectively; these values were significantly lower than the percentage of live bacteria observed after toothbrushing with sterile water (P=0.002, P=0.003 and P<0.001, respectively). The details of these results are presented in  Table 1.

**Table 1:**
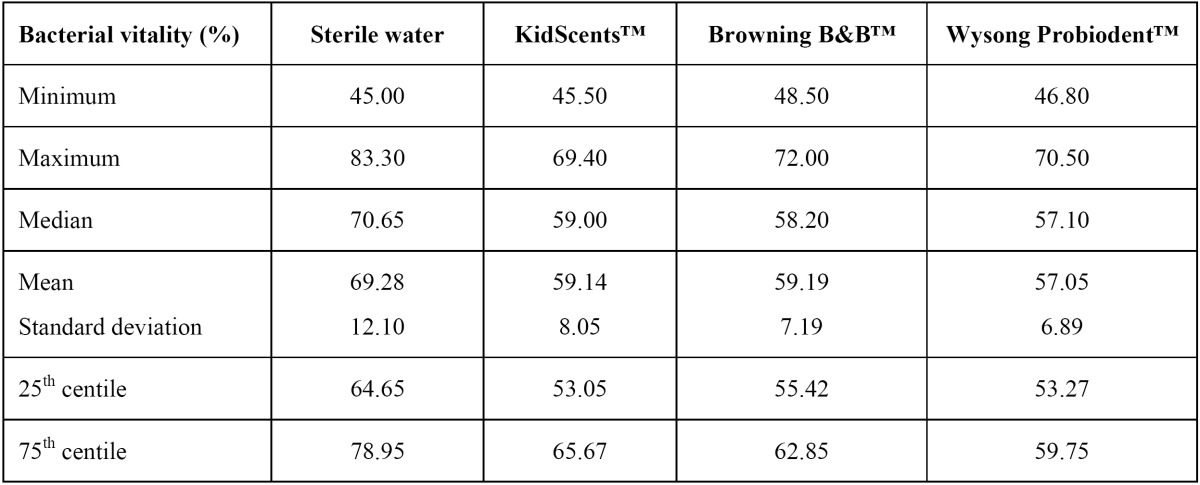
Percentage of live bacteria detected in the saliva samples after toothbrushing with sterile water or with the KidScents™, Browning B&B™ or Wysong Probiodent™ edible toothpastes.

On evaluation of newly formed dental plaque using the Turesky-Quigley-Hein plaque index (8) 24 hours after toothbrushing, the minimum value was observed with sterile water (plaque index=0.690) and the maximum value with the Wysong Probiodent™ toothpaste (plaque index=2.200). The mean values of post-toothbrushing plaque accumulation with the KidScents™, Browning B&B™ and Wysong Probiodent™ toothpastes were 1.352, 1.369 and 1.693, respectively ( Table 2), all of which were significantly higher than the value obtained after toothbrushing with water (mean=1.163; P=0.047, P=0.032 and P<0.001, respectively). Plaque regrowth was greater after toothbrushing with Wysong Probiodent™ toothpaste than after using KidScents™ (P=0.001) or Browning B&B™ (P=0.001) toothpastes.

**Table 2:**
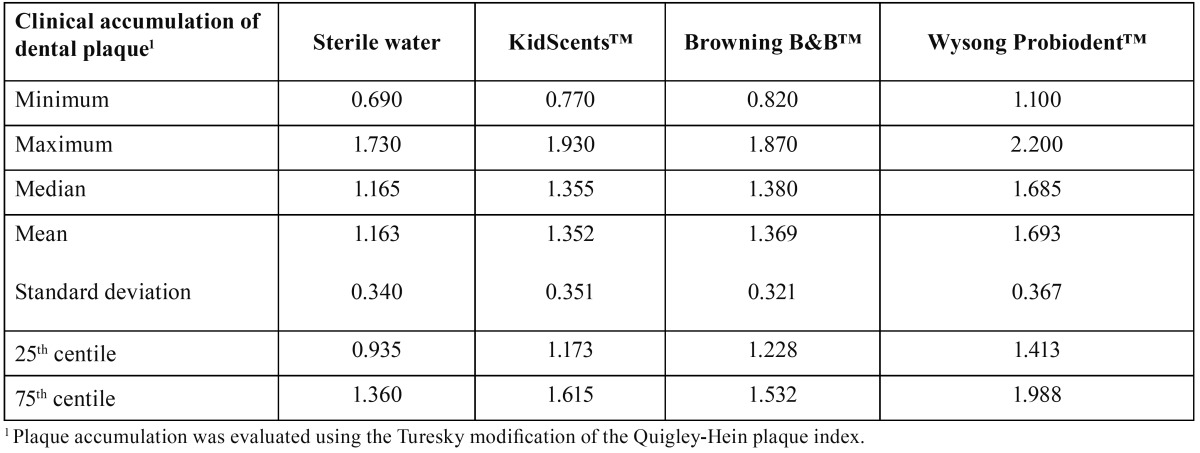
Dental plaque regrowth 24 hours after toothbrushing with sterile water or with the KidScents™, Browning B&B™ or Wysong Probiodent™ edible toothpastes.

## Discussion

The most widely used design in studies of plaque regrowth published in the literature is the so-called “four-day plaque regrowth” (9,10) or its variants (11). However, in this study it was decided to evaluate plaque regrowth over a shorter period (24 hours) after a single application of each one of the edible toothpastes selected. This situation of short-term plaque regrowth that we have used is more relevant to daily life, and its efficacy has already been demonstrated for the evaluation of potential plaque inhibitor agents and new active substances (12). The volume of toothpaste used (0.4 ml) was based on an estimation made by the authors of the quantity of toothpaste that an adult should use for conventional toothbrushing. The quantification of viable salivary bacterial populations was performed by epifluorescence microscopy with specific fluorochromes. The advantages of this method include that it is a rapid technique that is easy to perform, it quantifies bacterial viability in real-time (13), the SYTO 9/YP solution simultaneously identifies viable and non-viable bacteria (13), and it is possible to detect bacteria that cannot be cultured using plate techniques (14). Many studies on the quantification of dental plaque have used subjective indices based on the criterion of a clinical examiner (15). We therefore used the Turesky modification of the Quigley-Hein plaque index (8), as this is the most widely applied index for the evaluation of dental plaque (9,16,11).

The three toothpastes evaluated in this study present greater antibacterial activity than the control toothbrushing with sterile water. In general, commercial toothpastes incorporate an agent with antimicrobial activity and produce a fall in the bacterial counts of aerobes, anaerobes and *Streptococcus* spp.; the most significant reductions are observed with toothpastes containing antiseptics, particularly chlorhexidine (17). With the increase in the prevalence of antimicrobial resistance, attention is currently being focused on the use of natural antimicrobial components (18). The toothpastes we have tested include products such as essential oils (KidScents™), natural plant extracts (Browning B&B™), and probiotics and enzymes (Wysong Probiodent™). Some of these components, such as *Mentha spicata*, have powerful antibacterial activity against microorganisms responsible for the formation of dental plaque, such as *Streptococcus mutans* and *Streptococcus pyogenes* (19). Clove oil (*Syzygium aromaticum*) (KidScents™) has also been shown to be effective against gram-negative anaerobic periodontal pathogens such as *Porphyromonas gingivalis* and *Prevotella intermedia* (20). The majority of these agents have a broad spectrum of antibacterial activity and their prolonged use does not therefore alter the natural balance of the oral microbiota nor does it favour the appearance of microbial resistance (21). Another important characteristic of the toothpastes tested is their substantivity, as the time that their antimicrobial activity persists is not known; with many conventional toothpastes, this activity does not persist for more than five hours, which is the minimum period necessary for clinical determination of antiplaque and anti-gingivitis efficacy (22).

The inhibition of plaque regrowth has previously been demonstrated in a 24-hour model using toothpastes with chlorhexidine and fluoride (12). In the present study, in contrast, greater regrowth was observed with the three toothpastes tested than after toothbrushing with water. It has been suggested that the majority of individuals only eliminate 40% of dental plaque in a single toothbrushing session, and that the remaining plaque is responsible for the rapid regrowth (23). Because of this, plaque regrowth in studies that have quantified dental plaque before and after toothbrushing may have been conditioned by a deficient tooth brushing technique or by the methodological design of those studies (24-26). In the present study, in order to minimise these biases, the participants were selected from among dental students (presumably with a good toothbrushing technique) and, furthermore, the level of plaque on starting the experiment was zero in all cases; the plaque accumulated at 24 hours could therefore be attributed to the fact that the toothpastes evaluated lacked antiplaque activity. Some toothpastes that incorporate the main components of the toothpastes evaluated in the present study do have antiplaque and anti-gingivitis activity. The clinical efficacy of this activity has been demonstrated in toothpastes with essential oils (27,28) and with medicinal plants (29,30), though the inhibition of plaque regrowth after the application of toothpastes with probiotics has still not been adequately evaluated. As a result, we have confirmed that it cannot be assumed that a toothpaste is effective merely because it contains a known active agent, as that agent could be inactivated by other components in the formula. It has been suggested that if a toothpaste does not contribute to plaque inhibition, it does not necessarily mean that it should be withdrawn, as it may be a very effective vehicle for the delivery of fluoride (2); however, that argument is not applicable to edible toothpastes.

In conclusion, the results of this study allow us to state that the edible toothpastes tested have a degree of immediate antimicrobial activity but lack antiplaque activity, and even favour plaque regrowth in the short term.
